# Psychosocial deprivation and receptive language ability: a two-sample study

**DOI:** 10.1186/s11689-020-09341-2

**Published:** 2020-12-16

**Authors:** Kathryn L. Humphreys, Laura S. Machlin, Katherine L. Guyon-Harris, Charles A. Nelson, Nathan A. Fox, Charles H. Zeanah

**Affiliations:** 1 Vanderbilt University, Nashville, TN 37203 USA; 2 Tulane University School of Medicine, New Orleans, USA; 3 University of North Carolina, Chapel Hill, Chapel Hill, USA; 4 University of Pittsburgh School of Medicine, Pittsburgh, USA; 5 Boston Children’s Hospital/Harvard Medical School, Boston, USA; 6 Harvard Graduate School of Education, Cambridge, USA; 7 University of Maryland, College Park, USA

**Keywords:** Deprivation, Neglect, Receptive language, Socioeconomic status

## Abstract

**Background:**

The quality of early caregiving experiences is a known contributor to the quality of the language experiences young children receive. What is unknown is whether, and if so, how psychosocial deprivation early in life is associated with long-lasting receptive language outcomes.

**Methods:**

Two prospective longitudinal studies examining early psychosocial deprivation/neglect in different contexts (i.e., deprivation due to institutional care or deprivation experienced by children residing within US families) and receptive language as assessed via the Peabody Picture Vocabulary Test (PPVT) were used to assess the magnitude of these associations. First, 129 participants from the Bucharest Early Intervention Project, a randomized controlled trial of foster care as an alternative to institutional care in Romania, completed a receptive language assessment at age 18 years. Second, from the USA, 3342 participants from the Fragile Families and Child Wellbeing Study were assessed from infancy until middle childhood.

**Results:**

Children exposed to early institutional care, on average, had lower receptive language scores than their never institutionalized counterparts in late adolescence. While randomization to an early foster care intervention had no long-lasting association with PPVT scores, the duration of childhood exposure to institutional care was negatively associated with receptive language. Psychosocial deprivation in US families was also negatively associated with receptive language longitudinally, and this association remained statistically significant even after accounting for measures of socioeconomic status.

**Conclusion:**

Experiences of psychosocial deprivation may have long-lasting consequences for receptive language ability, extending to age 18 years. Psychosocial deprivation is an important prospective predictor of poorer receptive language.

**Trial registration:**

Bucharest Early Intervention Project ClinicalTrials.gov Identifier: NCT00747396

## Background

Children vary in the responsive and stimulating care that they receive, with some children receiving highly emotionally and cognitively enriched environments, whereas others experience significant deprivation in the form of psychosocial interactions. Psychosocial deprivation is associated with a number of negative outcomes across domains [26, 35, 41]. Among these deficits, seminal work examining the functioning of children from settings characterized by psychosocial deprivation (e.g., orphanage care) indicates that deprivation is associated with reduced language ability, with notable difficulties in receptive language ability (i.e., the ability to understand words or sentences) [20, 27, 44, 54]. Youth who have been institutionalized have receptive language scores well below the mean performance even after they no longer reside in an institution [8, 57, 58]. Thus, work from institutionalized samples suggests that there may be prolonged effects of early neglect even following placement into high-quality family care. Importantly, not all family-based settings are characterized as highly enriching, as variation in young children’s environments exists along a continuum [29]. As such, children residing in institutional care as well as home-based settings can be characterized along a dimension of stimulation/psychosocial deprivation.

Receptive language is necessary for facilitating successful communication. Across development, acquisition of receptive language skills typically precedes the development of expressive language skills [32]. Early in life, receptive language skills develop rapidly with great variability between children, followed by a relative slowing of receptive language development in middle childhood [2, 47, 53]. The optimal development of receptive language skills in early childhood is associated with a cascade of numerous positive outcomes across childhood such as better-developed language production and reading skills and higher emotional competence [3, 23, 49]. Individuals who have difficulties with receptive language may find it challenging to understand others during communication exchanges. In fact, deficits in receptive language ability early in life have been associated with social difficulties in adolescence including poor friendship quality [16, 50]. Thus, understanding factors associated with difficulties may help to identify those most at risk for not only poorer language and communication functioning, but also social-emotional development.

Though data specific to deprivation are limited, maltreatment has been linked to difficulties in receptive language ability. In a meta-analysis of nine studies that examined receptive language in children with and without maltreatment exposure, Lum et al. [34] found a medium effect (standardized mean difference = 0.53 [0.22, 0.84]) such that nonmaltreated children had higher scores than maltreated children. Importantly, these studies included a nonmaltreated group matched to the maltreated group in demographic characteristics, reducing the likelihood that these patterns could be explained by factors such as socioeconomic status (SES).

The purpose of the present study is to extend prior work examining the link between psychosocial deprivation and receptive language. Specifically, we aimed to leverage two longitudinal samples, one examining children with varying institutional care exposure and another from a population-based birth cohort of children in the USA with assessments capturing the variation in psychosocial neglect in early life. First, using data from the Bucharest Early Intervention Project (BEIP; study 1), we sought to examine receptive language ability as a function of the following: (a) children with and without exposure to institutional care, (b) among institutionalized children, randomization to a high-quality foster care, and (c) the amount of time spent in institutional care. The last assessment of language outcomes from the BEIP occurred when children were age 8 years (approximately 10 years prior to the outcomes reported here) and included expressive but not receptive language [58].

In a second study, drawn from participants enrolled in the Fragile Families and Child Wellbeing Study (FFCWS; study 2), we sought to examine whether variation in psychosocial neglect in family contexts is associated with receptive language in middle childhood. Importantly, both study 1 and study 2 samples assess receptive language using the same measure (i.e., the Peabody Picture Vocabulary Test). An ongoing area of interest in the study of early adversity is how best to determine potential specificity of associations when risk factors are correlated (children exposed to lower SES households may also be at higher risk for experiencing neglect or stress [13]). Given that SES has been linked to receptive language outcomes [56], in analyses from study 2 we examined the association of variation in early deprivation with receptive language ability after accounting for differences in SES.

## Study 1

### Methods

#### Sample

**Fig. 1:**
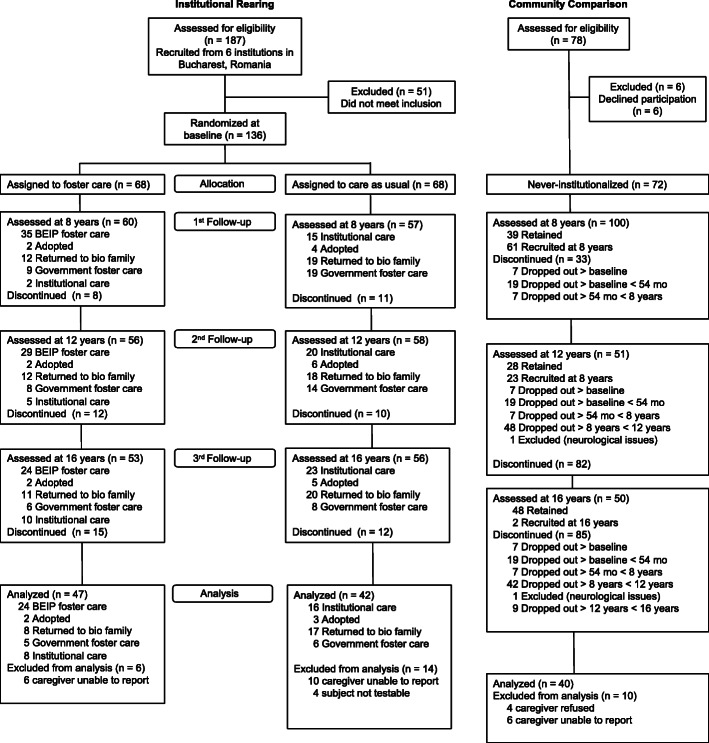
PRISMA for study flow from the Bucharest Early Intervention Project. Childhood deprivation experiences and receptive language were assessed as part 5 of the 3rd Follow-up/age 16 year assessment (when participants were approximately age 18 years)

The participants in this investigation were 129 individuals (69 female, 60 male) who were assessed at a mean age of 18.74 years (*SD =* 0.65) as part of the BEIP study. The BEIP is the first ever randomized controlled trial of foster care as an alternative to institutional care [62]. Children between the ages of 6 and 31 months were assessed at baseline and, following meeting inclusion criteria, were randomized either to high-quality foster care or to care as usual (typically resulting in greater duration of exposure to institutional care). The study team recruited never institutionalized community comparison children and conducted assessments of all groups at ages 30, 42, and 54 months, at which time the trial ended and supervision of the foster care network was taken over by local government authorities. Additional follow-up assessments were conducted at age 8, 12, and 16 years. The present study is part of the age 16 year follow-up, although children were 18 years old at the time of the receptive language assessment as this visit was added following the initiation of the age 16 data collection. Of the original 136 children, 89 were included in this follow-up (Fig. 1). The remaining 40 children were a never institutionalized group (NIG) of Romanian children recruited from area hospitals or schools in Bucharest who were included as a typically developing comparison group. The majority of participants (*n* = 87; 67%) were Romanian in ethnicity, 30 (23%) were Rroma, and 12 (9%) had another or unknown ethnicity.

Following approvals by the institutional review boards of the three principal investigators (NAF, CAN, CHZ) and by the local Commissions on Child Protection in Bucharest, the study commenced in collaboration with the Institute of Maternal and Child Health of the Romanian Ministry of Health. A data safety monitoring board in Bucharest reviewed the assessments for the current follow-up. As part of the longitudinal follow-up of these individuals from childhood, consent was obtained and signed for by each child’s legal guardian. In addition, written [or verbal] informed consent was obtained from all participants. Ethical considerations are discussed in detail elsewhere [38, 61]. All procedures contributing to this work comply with the ethical standards of the relevant national and institutional committees on human experimentation and with the Helsinki Declaration of 1975, as revised in 2008.

#### Randomization and masking

Following baseline assessments, children were randomly assigned to care as usual (care as usual group [CAUG]) or to foster care (foster care group [FCG]) by drawing participant numbers from a hat. Following randomization, the study had a non-interference policy and placement decisions were made by Romanian child protection authorities. In the years following randomization, in addition to some CAUG children obtaining family placements, some FCG children were returned to the parents who had abandoned them, some children were placed in later-emerging government foster care, and some were later readmitted to institutions (see Fig. 1). At the assessment in which receptive language was obtained, the intent-to-treat groups comprised 42 children in the CAUG and 47 children in the FCG. As noted above, there were also 40 comparison children in the NIG.

#### Measures

##### Receptive language ability

At age 18 years, participants were administered the Peabody Picture Vocabulary Test (PPVT-4), a common norm-referenced assessment of receptive language ability [15]. The PPVT was translated into Romanian, back-translated into English, and assessed for meaning at each step by bilingual research staff. Romanian words paired with each item from the PPVT-4 are available from the study team upon request (http://www.bucharestearlyinterventionproject.org/). The standardized score was used to best reflect the performance of the individuals relative to same-aged peers.

#### Data analytic plan

Standard scores from the PPVT were obtained for each group. Analysis of variance tests were used to examine differences between groups (ever vs. never institutionalized; foster care vs. care as usual assignment). *F* values, degrees of freedom, and *p* values along with 95% confidence intervals (CIs) of group differences are presented from ANOVAs, covarying participant sex. Linear regression was used to examine percent time in institutional care through age 18 years as a predictor of standard scores from the PPVT. We evaluated sex as a potential predictor of PPVT scores and included this as a covariate given a trend level association when considered in the full sample (see below).

### Results

#### Institutional care history

We first probed potential differences between children with and without exposure to institutional care. A significant group difference was found, *F*(1, 226) = 35.39, *p* < .001, such that the NIG (M = 105.42, SE = 2.58) had higher PPVT standard scores than those ever institutionalized (M = 86.88, SE = 1.72) with a large effect (Cohen’s *d* = 1.14 [0.73, 1.52]). Participant sex did not reach statistical significance, *F*(1, 226) = 3.75, *p* = .055.

#### Intent-to-treat analyses

The same analytic approach was used to examine whether those ever institutionalized children differed based on assignment to foster care or care as usual. There were no significant group differences based on initial group assignment in receptive language ability at age 18 years, *F*(1, 86) = 1.93, *p* = .168, such that the FCG (M = 89.34, SE = 2.64) did not significantly differ in PPVT standard scores from the CAUG (M = 84.34, SE = 2.79) with a small effect (*d* = 0.29 [− 0.13, 0.71]). Participant sex did not reach statistical significance, *F*(1, 86) = 1.76, *p* = .189.

#### Percent time in institutional care exposure

**Fig. 2:**
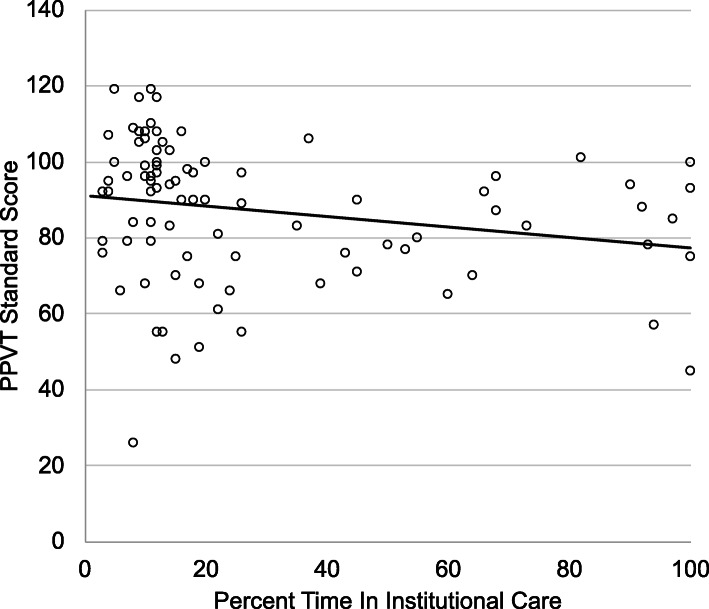
Scatterplot illustrating the association between percent time in institutional care from birth to age 18 years and standard score from the Peabody Picture Vocabulary Test (PPVT) at age 18 years. Raw associations are presented and statistics provided in the text are adjusted for participant sex

Last, given numerous placement changes that resulted in differential exposure to institutional care, we examined receptive language ability as a function of the percent of time individuals spent in institutional care from birth to age 18 years (see Fig. 2). Specifically, after covarying for sex, greater percent time in care was a significant predictor of lower receptive language ability at age 18 years (*β* = − .23, *t*(86) = − 2.26, *p* = .026). Percent time in institutional care explained a significant proportion of variance in PPVT scores, Δ*R*^2^ = .06.

As an exploratory analysis, we examined whether the age of placement into foster care was associated with receptive language among the FCG, but it was not (*β* = .04, *t*(43) = 0.12, *p* = .891, Δ*R*^2^ = .002).

## Study 2

### Methods

#### Sample

The Fragile Families and Child Wellbeing Study (FFCWS) followed a population-based, birth cohort of 4898 children born between 1998 and 2000 across 20 cities in the USA. FFCWS oversampled for nonmarital births and includes a diverse sample of children. Detailed recruitment and design data are available [46] with additional details at https://fragilefamilies.princeton.edu/documentation. At the age 9 follow-up assessment, 3630 parents completed the follow-up survey and 3346 children completed the Peabody Picture Vocabulary Test (PPVT-III) [14]. We included youth in the current sample if they had valid data on the PPVT from age 9 years. This resulted in a sample of 3346, 68% of the original sample. Data were missing from four children on maternal education for a final sample of 3342 children. A total of 1601 children were identified by their parent as female (48%). At year 1, both parents, if available, reported race/ethnicity, which were used to report on child’s race/ethnicity. In this sample, based on this approach, there were 519 White non-Hispanic/Latinx children (16%), 1642 Black/African American non-Hispanic children (49%), 827 Hispanic/Latinx children (25%), and 332 other non-Hispanic or multi-racial children (10%). Data from years 1, 3, and 9 are used in the current analysis.

#### Measures

##### Maternal education

Mother’s educational level collected at the beginning of the study (i.e., in year 1 when children were between the ages of 0 and 1 year) was coded 1 (less than high school) through 4 (college or graduate school).

##### Income-to-needs

Income-to-needs ratio was the total household income divided by the income threshold required for a family of that size, according to the Census Bureau at the age 1 follow-up assessment.

##### Deprivation

The latent construct of deprivation was modeled from six manifest indicators comprised of both mother- and observational-report data from the interviews conducted when participants were age 1 and 3 years. For detailed information about the development and analysis of the latent construct of deprivation, see previous work [36]. Here, we briefly describe each indicator (more information about indicators is publicly available information about FFCWS).

*Cognitive stimulation.* At year 1 and year 3, mothers reported how many days a week they sang nursery rhymes with their child, had their child complete household chores (year 3 survey only), played imaginary games with their child, read or told stories to their child, played with toys inside with their child, went out to eat with their child, or put their child to bed. The cutoffs for each were selected such that at least 5% of the sample and less than 15% of the sample would be identified in the “low” group. For example, for parents who reported playing imaginary games, going out to eat, and telling stories once a week or more, each of those experiences was coded as a 1. Parents reporting letting children help with household chores and reading stories twice a week or more were each coded as a 1. Parents who reported playing with toys inside three or more times a week were coded as a 1. Parents who reported singing nursery rhymes and putting a child to bed four or more times a week were each coded as a 1. Then, the sum of those variables resulted in the year 3 cognitive stimulation variable. With all the variables coded in this way (nursery rhymes, household chores, imaginary games, read stories, told stories, played with toys inside, went out to eat, put child to bed), most mothers reported the behavior frequently (54.3% of the sample engaged in every activity). The number of behaviors coded as present was summed and reverse scored such that higher scores represent greater deprivation. Item correlations are presented in supplemental table 1.

*Child books.* During the in-home interview at year 3, mothers were asked about how many books they had for their child. This variable was coded from 1 (5 or more books) to 4 (no books). Higher scores reflect fewer books (greater deprivation).

*Toys*. Mothers were asked how many toys their child had (e.g., push and pull toys, toys that can be put together in different ways, toys with wheels). These were coded as present or absent and summed for the total number of toys available to the child. This score was then reversed such that higher scores indicate represent greater deprivation.

*Positive parenting interactions*. During a home visit, observers documented if they observed any of seven possible positive social interactions between mother and child (e.g., “parent vocalized to child twice,” “parent responded to child’s vocalizations”). These were coded as present/absent by the observer; the number of absent variables was summed for each participant.

*Neglect*. During the home interview, mothers answered 19 items from the Parent–Child Conflict Tactics Scale [52]. Neglect is operationalized as the average for mother or other caregiver on the four items which assess neglect (e.g., not able to make sure child got to a doctor or hospital when needed). Higher scores reflect greater deprivation.

##### Receptive language ability

At the age 9 in-home interview, the child was administered Peabody Picture Vocabulary Test (PPVT-III) [14]. Here, we used the standard score which reflects the performance of the children relative to same-aged peers.

#### Data analytic plan

Data management and preliminary analyses were computed using SPSS 25 (IBM Statistics). Structural equation models (SEM) were conducted with Mplus 8.1 [40]. Across all models, model fit was evaluated using a variety of indices. The chi-square statistic is sensitive to the sample size, resulting in a tendency to reject models with large sample sizes. Thus, alternative model fit statistics are recommended [24]. A root mean square error of approximation (RMSEA) lower than 0.05, a comparative fit index (CFI) over 0.95, and a standardized root mean square residual (SRMR) under 0.08 indicate well-fitting models [24, 25]. All regression coefficients and factor weights are presented standardized. To account for continuous and categorical indicators in the deprivation latent construct, the full model used a robust weighted least squares (WLSMV) estimator [18].

**Fig. 3:**
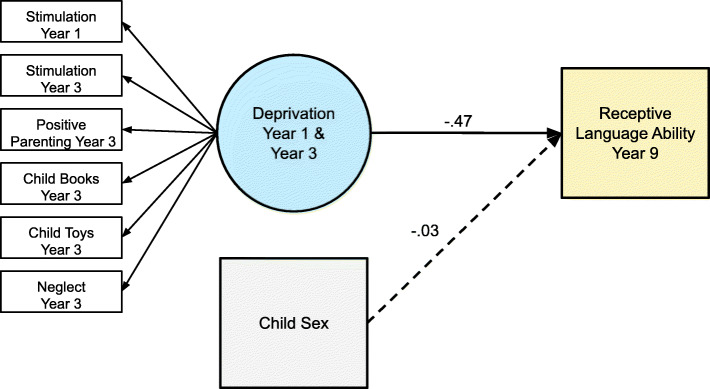
Model demonstrating associations between deprivation and receptive language ability of the child at year 9. Coefficients are standardized betas. Solid lines depict significant paths. Cognitive stimulation at years 1 and 3 was allowed to covary. For factor loadings of deprivation, see Table 1

We constructed measurement models of deprivation using confirmatory factor analysis (CFA) following standard SEM procedures [30]. To test our hypothesis, we tested the primary hypothesized structural model and examined fit (Fig. 3). In this model, deprivation predicted PPVT at age 9 years. Then, we additionally covaried for maternal education and income-to-needs ratio and tested the same hypothesized structural model. Finally, we tested a mediation model with the two predictors: maternal education and income-to-needs, with deprivation as the proposed mediator, and age 9 PPVT score as the outcome. This model included direct paths from deprivation, maternal education, and income-to-needs to age 9 PPVT. Additionally, deprivation was included as a mediator of the association between maternal education, income-to-needs, and PPVT score. Thus, the indirect paths from maternal education and income-to-needs to PPVT through deprivation were simultaneously estimated, which allows us to obtain estimates for each path over and above the other (i.e., the indirect effect of maternal education and receptive language through deprivation, after accounting for the indirect effect of income-to-needs and receptive language through deprivation, and vice versa). Maternal education and income-to-needs were allowed to covary. Indirect effects were estimated using a nonparametric, bias corrected bootstrapping approach with 1000 bootstrap re-samples; indirect paths are considered significant if the 95% confidence intervals do not contain zero [43]. We covaried for sex in all models.

### Results

#### Descriptive statistics

Basic descriptive information, including means, standard deviations, and item-level correlations, for each deprivation indicator can be found in Miller et al. [36]. Deprivation was negatively correlated with receptive language ability, *β* = − 0.47, *p* < .001.

#### Measurement model

The measurement model for the deprivation latent construct demonstrated good fit, *χ*^2^ = 60.33, *p* < .001, RMSEA = .03 [.019, .034], CFI = 0.95, SRMR = 0.04. All indicators loaded positively and significantly onto the deprivation latent construct (Table 1).

**Table 1:** Standardized deprivation latent factor loadings for main structural model

	Age	Factor loading	Factor loading SE	*p* value
**Deprivation**				
Cognitive stimulation	1	.32	.03	< .001
Neglect	3	.15	.02	< .001
Number of toys in the home	3	.55	.03	< .001
Number of child books	3	.70	.04	< .001
Positive parenting interactions	3	.47	.04	< .001
Cognitive stimulation	3	.31	.03	< .001

For the Deprivation construct, cognitive stimulation at years 1 and 3 was allowed to covary

#### Main structural model

The structural model demonstrated good fit to the data, *χ*^2^ = 51.34, *p* < .001, RMSEA = .02 [.02–.03], CFI = 0.97, SRMR = 0.02. Greater deprivation was associated with lower receptive language ability, *β* = − 0.47, *p* < .001. Sex was not significantly associated with receptive language ability, *β* = − 0.03, *p* = .091. Overall, the structural model accounted for 22% of the variance in receptive language ability (*p* < .001).

#### Covarying for socioeconomic status

When additionally controlling for maternal education and income-to-needs, the model demonstrated good fit, *χ*^2^ = 72.63, *p* < .001, RMSEA = .02 [.01–.03], CFI = 0.99, SRMR = 0.02, after allowing for maternal education and income-to-needs to covary. Greater deprivation was significantly associated with lower receptive language ability, *β* = − 0.29, *p* < .001. Maternal education (*β* = 0.18, *p* < .001) and income-to-needs (*β* = 0.14, *p* < .001) were each also significantly associated with higher receptive language ability. Sex did not significantly predict receptive language (*β* = − 0.03, *p* = .091). This model accounted for 26% of the variance in receptive language ability (*p* < .001).

#### Exploratory models

We explored the potential for an indirect effect from higher maternal education to higher receptive language ability through lower deprivation, and found this pathway to be statistically significant, *β* = 0.09, 95% CI 0.06–0.13. Further, the indirect effect from higher income-to-needs to higher receptive language ability via lower deprivation also was statistically significant, *β* = 0.10, 95% CI 0.06–0.15. These indicate that each predictor has unique variance explained in their associations with receptive language through deprivation.

## General discussion

In the present study, we examined the prospective association between psychosocial deprivation and receptive language in two different samples (i.e., the BEIP and the FFCWS). In the BEIP, individuals in late adolescence with childhood exposure to institutional care had, on average, substantially lower receptive language ability compared to those who had never been institutionalized. Furthermore, among those with a history of institutional care, more time spent in institutional care from birth to age 18 years was associated with poorer receptive language ability at age 18 years. In the US-based FFCWS study, children were followed from early life through middle childhood, and variation in neglect in the first years of life was associated with poorer receptive language outcomes at age 9 years. Despite clear differences in the operationalization of deprivation across the two studies, we found evidence that higher levels of deprivation were associated with poorer receptive language outcomes. Language learning is thought to require frequent opportunities for child-directed speech and communicative interactions [31, 55], and given the high child–caregiver ratios [12], children in institutional care are less likely to receive input that would promote responsive interactions. Responsive interactions also vary in family care contexts. In fact, one prior study [51] directly compared the language outcomes of 15 children with a history of institutionalization and 17 children with a history of familial neglect in the USA. These groups did not differ in receptive language ability, although both groups performed worse than community children. The findings from the FFCWS study provide a conceptual replication of this work, such that exposure to deprivation in early life may have lasting consequences for receptive language ability.

Across middle childhood and adolescence, children enter into increasingly complex peer relationships, which are dependent upon reciprocal communication that draws heavily on both receptive and expressive language skills [7, 19]. Receptive language is important for social-emotional development including the development of social skills, quality friendships, and emotional competence [3, 16], particularly when accompanied by deficits in expressive language [50]. Further, receptive language predicts externalizing behavior [59, 60], including as a mediator of the link between deprivation and externalizing psychopathology [37].

In addition to the association between deprivation and receptive language, the findings from the FFCWS help to clarify the role of SES in the pattern of this association. Specifically, deprivation was found to be a significant predictor of receptive language even after accounting for variation in maternal education level and income-to-needs ratio. Thus, across both samples, there is evidence for the role of environmental experiences in receptive language development. From the BEIP, the lack of group differences using an intent-to-treat approach somewhat weakens the ability to draw strong causal conclusions, yet the placement changes from the original group assignments may explain the fade-out effects specific to the intervention [57, 58]. There have been numerous placement changes in the time since placement (age 22 months) and since the official support for the intervention by the study team ended (age 54 months) (see [1]). Many children assigned to the care as usual condition were eventually placed in family care, and many children assigned to the high-quality foster care intervention experienced placement disruption which, in some cases, led to a return to institutional care.

Institutional care exposure is associated with numerous difficulties across domains, including in receptive language [4]. In a study of children adopted around age 3 years, approximately two thirds of children adopted from countries associated with the former Soviet Union were given a diagnosis of communication disorder [6], and no differences were found based on age of adoption. However, two other studies indicated that duration of exposure to institutional care may be important for receptive language outcomes. First, previously institutionalized children adopted by families in the USA found that duration of institutional care exposure was associated with poorer performance on standardized language assessments, specifically paragraph comprehension from the Comprehensive Assessment of Spoken Language and greater difficulties in understanding and following directions from the Clinical Evaluation of Language Fundamentals [33]. Second, in outcomes from the English and Romanian Adoptees Study, children exposed to very little institutional care (i.e., adopted prior to age 6 months) showed no differences compared to children adopted from within the UK in terms of receptive language outcomes [9]. Children adopted after this age were more likely to have language delays, but there was no association between duration of institutional care and language outcomes, indicating a nonlinear pattern linking duration of psychosocial neglect and language outcomes.

Importantly, we explicitly considered the role of SES in the FFCWS sample. Some have called for statistical control of SES in examining the association between maltreatment and language skills [34] given the link between SES and language [17, 21]. We find evidence of the independent associations of deprivation and measures of SES in relation to receptive language, and that the model with SES markers predicts only an additional 6% of the variance after accounting for deprivation. In exploratory analyses, we find preliminary evidence that variations in SES may be meaningful predictors of receptive language, in part, through variation in the psychosocial deprivation of the child’s environment. These findings are consistent with prior work [22] finding that environmental features mediate the association between SES and language outcomes. More work is needed to determine the potential interactive associations between deprivation and other family characteristics.

Different types of maltreatment frequently co-occur [11], making it difficult in most cases to isolate the potential unique contributions of threatening experiences and psychosocial deprivation experiences on child functioning. Findings from a study that compared children who experienced neglect but either did or did not have posttraumatic stress disorder (PTSD), as well as demographically matched comparison children without a history of abuse, found that children who experienced neglect but did not have a PTSD diagnosis had lower receptive language scores than comparison youth and neglected groups did not significantly differ from one another [10]. Such findings indicate that it may be psychosocial deprivation, rather than threat-related experiences, that is associated with receptive language. Exploring the potential specificity and differential effects of deprivation and threat on receptive language remains an important area for future work. Given that children from the BEIP are believed to have relatively low exposure to other forms of maltreatment, this study provides what is likely to be a purer case for examining the link between psychosocial deprivation and receptive language without the potential confounding of physical abuse or witnessing family violence.

This study has several limitations; each indicates promising avenues for future research. First, we focused solely on receptive language as the outcome of interest, despite many alternative measures of language ability. It may be that the form of language difficulties differs depending on the assessment used to study language domains. Interestingly, there is mixed evidence regarding whether receptive or expressive language may be less spared by early adversity [48, 57, 58]. Second, we were unable to thoroughly examine alternative adverse exposures (anesthesia [28]) to better isolate psychosocial deprivation from other potentially confounding influences. Other factors have been identified elsewhere, including parents’ marital status and number of children in the household which explained additional variance over and above the association of maternal education, indicating that other features of the children’s environment may be related to language exposure and communication with other adults are likely important for explaining variation even among children from low-income families [45]. Prenatal factors, unassessed in these studies, have also been posited as an explanation for poorer language outcomes [42]. While this study cannot explore this directly, the evidence from the BEIP in which children’s placements were at least in part randomly determined provides strong evidence for the role of postnatal environments in language outcomes.

## Conclusions

The present findings add to a growing literature tracing the long-term outcomes of children who experience varying levels of deprivation. Specifically, in two different samples, we find evidence of the prospective association between psychosocial deprivation/neglect, first in institutional care and then in family contexts, and poorer receptive language ability. Among the BEIP sample, we find no evidence of “catch up” to typical functioning when individuals reach early adulthood. These findings provide additional support for efforts to reduce children’s exposure to institutional care (see [5]). Among the FFCWS sample, we find that variation in neglect is a prospective predictor of receptive language outcomes even after accounting for SES. While there is preliminary evidence that deprivation may be a potential pathway explaining the significant association between SES and child receptive language, more research is needed to identify whether family characteristics would be a useful guide for targeted intervention to reduce risk for neglect. While we no longer find strong evidence of the high-quality caregiving intervention on receptive language outcomes in late adolescence from the BEIP, it is important to note that other studies have found support for the hypothesis that receptive language can be improved via intervention. Children who participated in a preschool program showed improvements in receptive language that persisted when assessed at age 10 years, indicating the promise of early intervention and persistence of effects over time [39].

## Supplementary Information

**Additional file 1:** **Supplemental Table 1**

## Data Availability

Data for the FFCWS can be found at fragilefamilies.princeton.edu/documentation.
