# Supplementary material for: Psychosocial deprivation and receptive language ability: a two-sample study
Source: J Neurodev Disord. 2020 Dec 16;12:36. doi: 10.1186/s11689-020-09341-2 (PMC7745465; doi:10.1186/s11689-020-09341-2)
Supplement: Supplementary file 1 — Additional file 1: Supplemental Table 1 [file 11689_2020_9341_MOESM1_ESM.docx]

| Variable | *M* | *SD* | 1 | 2 | 3 | 4 | 5 | 6 |
| --- | --- | --- | --- | --- | --- | --- | --- | --- |
| 1. Year 3 Neglect | 0.09 | 0.34 | – |  |  |  |  |  |
| 2. Year 3 Number of Toys in the Home | 1.44 | 0.85 | .07** | – |  |  |  |  |
| 3. Year 3 Number of Child Books | 1.14 | 0.47 | .06** | .26** | – |  |  |  |
| 4. Year 3 Positive Parenting Interactions | 0.94 | 1.51 | .11** | .15** | .16** | – |  |  |
| 5. Year 3 Cognitive Stimulation | 0.81 | 1.21 | .06** | .15** | .15** | .11** | – |  |
| 6. Year 1 Cognitive Stimulation | 0.97 | 1.39 | .09** | .09** | .09** | .15** | .27** | – |

Supplemental Table 1

*Means, standard deviations, and correlations (N=3,346)*
